# Reductionist methodology and the ambiguity of the categories of race and ethnicity in biomedical research: an exploratory study of recent evidence

**DOI:** 10.1007/s11019-022-10122-y

**Published:** 2022-11-09

**Authors:** Joanna K. Malinowska, Tomasz Żuradzki

**Affiliations:** 1 Faculty of Philosophy, Adam Mickiewicz University, Ul. Szamarzewskiego 89C, 60-568 Poznań, Poland; 2 Institute of Philosophy & Interdisciplinary Centre for Ethics, Jagiellonian University, Ul. Grodzka 52, 31-044 Kraków, Poland

**Keywords:** Biomedical research, Race, Ethnicity, Methodology, Genetics, Genomics, Reductionism, Covid-19

## Abstract

In this article, we analyse how researchers use the categories of race and ethnicity with reference to genetics and genomics. We show that there is still considerable conceptual “messiness” (despite the wide-ranging and popular debate on the subject) when it comes to the use of ethnoracial categories in genetics and genomics that among other things makes it difficult to properly compare and interpret research using ethnoracial categories, as well as draw conclusions from them. Finally, we briefly reconstruct some of the biases of reductionism to which geneticists (as well as other researchers referring to genetic methods and explanations) are particularly exposed to, and we analyse the problem in the context of the biologization of ethnoracial categories. Our work constitutes a novel, in-depth contribution to the debate about reporting race and ethnicity in biomedical and health research. First, we reconstruct the theoretical background assumptions about racial ontology which researchers implicitly presume in their studies with the aid of a sample of recent papers published in medical journals about COVID-19. Secondly, we use the typology of the biases of reductionism to the problem of biologization of ethnoracial categories with reference to genetics and genomics.

**Supplementary Information:**

The online version contains supplementary material available at 10.1007/s11019-022-10122-y.

## Introduction

Many scholars agree that the distinction between different races and ethnicities is not based on biologically relevant features but should be specified in terms of nonbiological social kinds. This approach is also clearly discernible in the latest guidelines on reporting race and ethnicity in medical and science journals (e.g., Flanagin et al. 2021). However, in practice, these categories still frequently surface in scholarly papers within medicine and health sciences as if they were determinants of innate, universal, and to some extent, essential traits of biomedical relevance (cf. Bhala et al. 2020; Hooper et al. 2020; Karaca-Mandic et al. 2021). The inclusion of ethnoracial categories as reference classes to recruit, analyse, and report research involving human participants is expected to lead to the reduction of knowledge gaps when it comes to populations previously underrepresented in research (in particular, the aetiology of health, disease, and response to drug treatment), and to provide data that may help to eliminate some health inequalities.

However, some authors have argued that folk racial classifications (especially those recognised in the relevant American regulations and based on self-declared racial identifications) can be epistemically useful in biomedical research and healthcare as there are some medically relevant (including genetic) differences between the continental populations they allegedly describe (Risch et al. 2002; Rosenberg et al. 2002; Burchard et al., 2003; Spencer 2018b). Moreover, some minority health advocates have urged decision-makers to require scientists to include members of various ethnoracial groups generally considered to have been underrepresented in previous clinical studies and to examine differences across groups with regard to treatment effects.

There are voices that these “racial differences” (even if resulting from social factors) should influence how researchers design, conduct, and interpret research with human subjects. Although the problem has been discussed in the US for decades, it was recently revived during the COVID-19 pandemic. For example, the authors of a commentary in Jama, discussing COVID-19 and racial/ethnic disparities during the early phase of the COVID-19 pandemic, wrote in May 2020: “The possibility that genetic or other biological factors may predispose individuals to more severe disease and higher mortality related to COVID-19 is an empirical question that needs to be addressed” (Hooper et al. 2020). This is a clear case of scientists referring to racial/ethnic disparities based on genetic or other biological factors, although they have no hypotheses as to how to connect this type of categorisation with any genetic or biological mechanisms that might explain such alleged interracial health differences. Analogical voices are concerned about the ways policymakers should design the allocation of scarce healthcare resources, such as the situation posed during the COVID-19 pandemic. Some organisations (e.g., The National Academies of Sciences, Engineering, and Medicine – NASEM) and scholars suggested prioritising access to scarce medical resources, e.g., intensive care units (ICU) during pandemics or COVID-19 vaccines in early 2021 based on racial or semi-racial (e.g., the Area Deprivation Index) categories (Schmidt et al. 2020; White and Lo 2021).

However, there are arguments (Perez-Rodriguez & de la Fuente, 2017; Hochman 2019, 2021a; Malinowska 2021) that the very use of the category of race and ethnicity in medical research and healthcare might lead to the unfounded reinforcement of the view that (especially when applied in genetics and genomics) significant biological and/or genetic differences exist between several large groups of people that correspond to folk racial divisions. The reason for that may be a popular bias (both among researchers and the public) of genetic essentialism, i.e., the idea that all (or most) differences in the characteristics of peoples (e.g., their behaviour, appearance, or health condition) can be analysed and explained by studying genetic variations between them (Dar-Nimrod et al. 2021; Popejoy 2021). Researchers in the fields of genomics and genetics are particularly prone to this type of essentialism due to their reductionist methodology^1^ i.e., “given that the field is fundamentally designed to overlook environmental or public health explanations in favour of identifying genetic variants that influence biological traits” (Popejoy 2021: 81). Acceptance of genetic essentialism might strengthen the tendency to biologize human races in science (Braun et al. 2007; Roberts 2011; Pollock 2012).

Reductionism is embedded in the methodologies of both classical genetics and genomics. It is a position which assumes that “the best strategy of research is to study living phenomena at the lowest levels of complexity” (Ayala 1987: 315). As a result of the tremendous progress and explanatory successes of molecular biology, genetics, and genomics, genes are precisely such a “lowest level of complexity”, not only for the representatives of these fields but also more generally for many researchers in the field of biomedical research (Tauber and Sarkar, 1992; Sarkar 1998, 2001). After the discovery of the structure and function of DNA, the vast majority of the members of these scientific communities began to expect that the investigation into the human genetic organisation and the molecular processes responsible for the gene’s expression would allow the explanation of all, or almost all, the issues related to the human ontogenetic development and treat all diseases considered to be genetic in origin (e.g., cancer or schizophrenia) (Joyner and Pedersen 2011). In the case of some research problems, the reductionist approach can be very useful and successful (e.g., in the case of rare Mendelian diseases, like Huntington’s chorea, where a single defective gene is associated with the development of pathologies (cf. Vonsattel and DiFiglia 1998)). However, a strong reductionist approach usually fails when dealing with more complex situations (e.g., for the analysis of many aspects of the COVID-19 pandemic, such as predicting the modulators of inflammation in patients, adjusting proper treatment, or developing new therapeutic drugs, other approaches, such as system biology, may be more fruitful cf. Hajjo and Tropsha 2020; Jaiswal et al. 2020; Jung et al. 2021). Although geneticists have significantly changed their perspective in recent decades (e.g., they have implemented models of complex traits that no longer rely on classical genetic reductionism) and many critical arguments have been formulated against the naïve application of radical reductionist methodology in biology or medicine (Tauber and Sarkar 1992; Bock and Goode 2008; Greene and Loscalzo 2017; Lerner, 2015), the reductionist approach is still quite common.

In this article, we analyse the use of the race and ethnicity category with reference to genetics and genomics in medical journals. We show that there is still considerable conceptual “messiness” (despite the wide-ranging and popular debate on the subject) that makes it difficult to properly compare and interpret research using ethnoracial categories in genetic contexts, as well as to draw conclusions from them.^2^ With the aid of a sample of recent papers published in medical journals about COVID-19, we also reconstruct the theoretical background assumptions about racial ontology that researchers implicitly presume in their studies. Finally, we reconstruct some of the biases of reductionism and analyse them in light of the biologization of racial categories in the studied articles.

## Categories of race and ethnicity in the articles on Covid-19 relating to genetics or genomics

### Method

The use of ethnoracial categories in medical and health sciences has been the subject of numerous studies that have focused on three main groups of questions: how researchers report on ethnoracial categories in their publications (Ma et al. 2007; Zhang and Finkelstein 2019; Maduka et al. 2021), on explanations/hypotheses for the use of ethnoracial categories in certain studies (Lee 2009; Sankar et al. 2007; Friedman and Lee 2013; Duello et al. 2021), and on how scientists conceptualise these categories (Lin and Kelsey, 2000; Huddart et al. 2019; Popejoy et al. 2020; Popejoy 2021). In the majority of such studies, researchers have adopted two methods to answer the above questions: broadly understood systematic reviews of the literature (e.g., content analysis) of published articles etc., or interviews and surveys carried out in a given community (e.g., geneticists). As philosophers, we decided to look at this problem from a different perspective. Although we used some quantitative procedures (e.g., to establish in how many papers the term “race” was used), the core of our method is strictly qualitative: conceptual analysis (Laurence and Margolis 2003) and conceptual review (Jesson et al. 2011: 79–80). Using conceptual analysis and review allowed us to reconstruct and compare how the authors operationalised ethnoracial categories even if they do not explicitly explain this in their papers. The analysis of the complete texts from our corpus gave us the opportunity to identify situations where explicit declarations about a particular interpretation of ethnoracial categories (e.g., as social constructs) did not fit with the content of their article (e.g., biologizing race).

We studied the full texts of 119 articles from 2020 and 2021 to understand how the terms “race”, “racial” or “ethnicity” co-exist in the biomedical literature about COVID-19 that used terms “gene”, “genetic”, “genomic”. Our aim was not to conduct a systematic review of the literature in the sense of assembling every article relevant to the research question but rather to have a sample of papers which would be representative of a specific area of interest within biomedicine that might illustrate the relevant ways in which race and ethnicities are used in that part of current medical research and to visualise reductionist biases. We intentionally decided to focus only on papers mentioning genetics/genomics because we wanted to see to what extent the biologization of race and ethnicities is still practised in this part of medical research, although the mainstream view clearly expressed a few years ago in the commentary published by the journal *Science* is quite the reverse: “racial classifications do not make sense in terms of genetics” (Yudell et al. 2016). Moreover, we concentrated on COVID-19, which is obviously not “a genetic” disease – although it is not impossible that genetic variations affect individual differences in susceptibility to severe forms of the disease. We selected the categories of race and ethnicity (not including e.g., ancestry) because these categories are most often found in various guidelines for the collection and use of demographic data (e.g., Office of Management and Budget guidelines for the US or The Census Order for England and Wales). However, we are also aware that this is changing, for example in the less official guidelines recently proposed by JAMA (Flanagin et al. 2021), where the term “ancestry” also appears. We also decided to only search within the metadata because our aim was to collect a corpus of papers that put the relevant terms (race, gene etc.) in a prominent place (article title, abstract, author-provided keywords) and was able to be carefully read by two persons. Repeating our search in a more comprehensive database (e.g., Google Scholar) returns many more results because such a database also covers: (i) many journals not indexed in PubMed; (ii) preprints; (iii) a default search covers full texts.

A search was carried out in the LitCovid database, which aims to precisely and comprehensively collect all of the relevant metadata (article title, abstract, author-provided keywords, authors) of articles related to COVID-19 in PubMed. LitCovid is “a curated literature hub for tracking up-to-date scientific information about the 2019 novel Coronavirus. It is the most comprehensive resource on the subject, providing central access to hundreds of thousands (and growing) relevant articles in PubMed”.^3^ LitCovid retrieves articles on a daily basis from PubMed, and “search results are then human-reviewed where relevant articles are identified and curated, with assistance from an automated machine-learning and text-classification algorithm”. PubMed is a large database (more than 34 million references) of biomedical literature.

Our first search on September 3, 2021, “(race OR racial OR ethnicity) AND (gene OR genetic OR genomic)”, generated 177 results within the metadata of papers indexed by the LitCovid database. Then, on September 9, 2021, we searched Web of Science (WoS) with the same search keywords (adding only “AND (Covid-19)” and searching through titles, abstracts, author keywords) to check the completeness of our previous LitCovid database search (some of the metadata in LitCovid was incomplete, e.g., missing abstracts). We added 11 papers that had not been found previously in LitCovid. Limiting the search to PubMed and WoS was intended to ensure that we obtained a corpus of papers published in high-quality journals in which editorial practices have already been verified and approved by The National Library of Medicine and WoS, respectively.^4^

The first step of the selection process involved screening titles and reading the abstracts. We excluded 49 publications because they did not concern race/ethnicity in a relevant sense (e.g., “A race against time” or “survival of the human race”). We also had to exclude 15 records for which we could not find a full text (e.g., because of incorrect links in LitCovid; published in journals inaccessible from the main university libraries or by any other method). In the final phase, which consisted of reading full papers, we excluded five more articles. Although they mentioned race/ethnicity in a relevant sense, they neither used racial/ethnic categories to interpret the results of empirical studies nor did they discuss them. Usually, they only referred to some other research on race/ ethnicity (e.g., “There have been many studies seeking to explore the correlations between COVID-19 clinical outcomes and various clinical variables, including age, sex, race…”). Then, we divided papers into three main formal categories that represented the main types of scientific communication in biomedical literature: (1) original empirical studies (68); (2) reviews and meta-analysis (44); (3) conceptual/theoretical papers, including editorials and commentaries (7).

In the next step, we analysed what racial terms (race, racial, ethnicity) the articles belonging to each of these groups contain and what these terms refer to. We determined how many used the terms “race” or “racial”, and how many only used the term “ethnicity” (including terms such as “ethnic group” or “ethnic population”). In most articles, ethnoracial categories were not explicitly defined or discussed. The conceptual review allowed us “to compare and contrast the different ways in which authors have used a specific word or concept” (Jesson et al. 2011: 79). To reconstruct what scientists referred to when using these categories in their papers, we studied the context in which they were used: research hypotheses, methodology, results (their interpretations and explanations) as well as the literature cited.^5^ We also analysed what racial categorisations researchers used (e.g., whether they are more precise classifications related to specific geographic populations or very naive and generic terms referring explicitly to folk racial beliefs).

After carefully and systematically reading all of the articles, we distinguished five different approaches to ethnoracial terms that appeared in the examined papers and labelled them as (a) folk, (b) demogeographic, (c) socio-cultural, (d) multileveled and (e) institutional (see: Table 1). This division is based on philosophical discussions concerning the ontology of race (cf. Glasgow et al. 2019; James and Burgos 2022). We studied various positions in this regard (e.g., population naturalism, constructivism, antirealism etc.) and then extended our analysis to include discussions on this topic that take place in the area of empirical sciences (e.g., Huddart et al. 2019). As a result, we distinguished four main ways of conceptualizing ethnoracial categories in the analysed articles (a–d). Additionally, we recognised that there was a further group of papers (e) in which ethnoracial categories are only used because of institutional recommendations and have no specific explanatory meaning. We defined all these approaches as:

- Folk: articles in which the category of race and/or ethnicity was used as a category indicating genetic differences between the representatives of the groups it differentiates. These distinctions are usually based on so-called “continental populations” and are more or less consistent with some of the institutional guidelines, especially in the US) e.g., Caucasians, African Americans and Asians. Often this position is related to the belief that there are some biological differences between humans, which the category of race aptly captures. They also largely correspond to folk ethnoracial classifications and use their terminology, e.g., Blacks and Whites (cf. Spencer 2018a, 2018b; Winsberg 2019).
- Demogeographic: articles in which the category of race and/or ethnicity was also used as a category indicating genetic differences between the representatives of the groups it differentiates. However, in contrast with a) this distinction does not reflect folk ethnoracial categories, like Blacks and Whites, or very broad continental divisions (like Asians and Caucasian). Demogeographic classifications try to capture people’s demographic or geographic origin more precisely, e.g., Amish, Latino, Ashkenazi Jewish, East Asian, Finnish etc. (cf. Huddart et al. 2019). Yet, without the authors defining these categories, it is unclear whether they are merely using them as proxies and recognising these proxies as constructs with a very limited epistemic value or accepting some form of population naturalism that interprets these proxies as “biologically informed” (Sesardic 2010).
- Socio-cultural: articles in which the category of race and/or ethnicity was used as indicating differences between the representatives of the groups it differentiates between based on solely social factors (cf. Appiah 1996; Zack 2014). Often this position implies the belief that biological human races do not exist but that races exist as social constructs that continue to influence the lives of people, including the condition of their health (e.g., due to systemic racism).
- Multileveled: articles in which the category of race and/or ethnicity was used as a category indicating some biologically relevant differences between the representatives of distinguished groups. However, these differences are due to the complex interactions of many factors, e.g., biological, social, and environmental (cf. Hochman 2019, 2021b; Malinowska and Żuradzki 2022).
- Institutional: articles in which the category of race and/or ethnicity was used because of the implementation of institutional guidelines (cf. Lee and Skrentny 2010; Kahn 2015; Flanagin et al. 2021), without any further analysis.

**Table 1:** Presents the results of an analysis of full texts of 119 articles containing the terms “race” or “racial” or “ethnicity” and “gene” or “genetic” or “genomic” found in LitCovid and Web of Science databases

The way of using racial categories	The type of scientific paper	Editorials and policy		Experimental		Reviews		Total
		Race	Ethnicity	Race	Ethnicity	Race	Ethnicity	
Folk		0	2	9	5	5	2	23
Demographic		0	0	2	30	4	12	48
Socio-cultural		2	1	3	3	5	0	14
Multileveled		1	0	1	5	8	3	18
Institutional		0	1	5	6	4	0	16
Total		3	4	20	49	26	17	119

We collected quotes representing each of the above positions (Table 2). The process of selection was intended to reflect their diversity and be representative of each of them. Due to the aforementioned lack of explicit definitions and conceptualisations of ethnoracial categories in the majority of analysed publications, we sought out quotations that most directly represented authors’ positions on how (in what sense) they use racial categories (in some cases also why they apply them) in their study. Finally, we analysed these quotations in the context of the reductionist biases characterised by Wimsatt (2006). We found that they may indicate an occurrence of some of these biases in regard to ethnoracial categories. At this point, it should be emphasized that while we referred to particular quotes, our aim was rather to investigate some general trends when it comes to the use of ethnoracial classifications with reference to genetics and genomics, and not to evaluate individual authors’ approaches to this issue.

**Table 2:** Examples of quotations of various interpretations, applications, and divisions of ethno racial categories appearing in the analysed articles

The manner in which racial categories are used	Article	Quote
**A. Folk**	A1 Li et al. 2020: 6	Third, the comparison of ACE2 expression levels between Asian and non-Asian races in TCGA datasets was performed in a small number of samples, and thus the associated results need to be verified in larger populations
	A2 Wambier et al. 2020:773	African Americans, as an ethnic group, tend to carry a shorter version of the CAG repeat in the androgen receptor gene (Bennett et al., 2002). Thus, AR polymorphisms could be a very important factor in the known ethnical vulnerability
	A3 Wambier et al. 2020: 771	The androgen sensitivity model explains why males are more likely to develop severe symptoms while children are ostensibly resistant to infection. Further, the model explains the difference in COVID-19 mortality rates among different ethnicities. Androgen sensitivity is determined by genetic variants of the androgen receptor
	A4 Hachim et al. 2020: 4	In light of the premise for a potential role for genetic susceptibility to cardiovascular injuries associated with COVID-19, we further explored for the expression of the identified DEGs in the publicly available dataset (GSE17078) of blood outgrowth endothelial cells from 27 healthy subjects of diverse ages and grouped into Caucasian and African Americans
	A5 Pathangey et. al 2021: 317–318	Increased ACE2: ACE2 expression is significantly higher among Asians than African Americans and Caucasians (326) Increased eQTLs: eQTLs associated with elevated ACE2 expression in tissues of Eastern Asian population: close to 100% in Eastern Asians and > 30% higher than other ethnic groups (268, 327) […]
	A6. Pathangey et. al 2021: 301	No difference in genetic polymorphisms: Asians and other races express similar levels of genetic polymorphisms of the SARS-CoV-2 entry receptor (328, 329) The secondary risk factors of age, sex, and race/ genetics had limited-to-moderate evidence
	A7 Abdelzaher et al. 2020: 7	Therefore, we can relate that races with high risk of thrombosis and the relation found between the pathogenesis of COVID-19 and coagulopathy show the possibility that those races are susceptible to COVID-19 mortality
**B. Demogeographic**	B.1 Wang et al. 2021: 309	In the current study, populations were divided into eight categories, on the basis of ethnic origin as described previously: African ethnic origin (AFR), Latino ethnic origin (AMR), EAS, South Asian ethnic origin (SAS), Finnish ethnic origin (FIN), non-Finnish ethnic origin (NFE), Ashkenazi Jewish ethnic origin (ASJ) and Other ethnic origin (OTH)
	B.2 Wang et al. 2021: 314	In addition, ethnic differences were analysed for these actionable pharmacogenes. We found that most functional variations of the three genes showed ethnic bias among populations. As indicated, Africans showed an obviously higher frequency than other populations for most of the mutations
	B.3 Wang et al. 2021: 316	Generally, drugs influenced by ethnicity-based differences in pharmacogenes should be studied first. As mentioned above, the differences in trials among different ethnic groups were determined in 132 reported COVID-19 studies (Fig. 2C). To comprehensively explore the ethnicity-based differences in pharmacogenes of all drugs in COVID-19 therapy, we employed the “Drug Score”. These scores were calculated based on the cumulative “Gene Score” deviations of all pharmacogenes between one ethnicity and seven other ethnic groups for each drug and represented the ethnicity-based differences of this drug compared with seven other ethnic groups
	B4 Biswas and Majumder 2020: 457	This will inform the relationship of coronavirus type with host ethnicity, perhaps mediated through differences in frequencies of variants in genes of the immune system among ethnic groups in India
	B5 Yamamoto et al.^2020^: 17	We discovered that a gene (ACE1) and its genotype (I/D) seem to provide a plausible explanation for why some ethnic groups, especially Europeans populations, have been more heavily affected by SARS-CoV-2 than Asians populations
	B6 Hubacek et al. 2021: 207	These discrepancies could be caused by the fact, that there could be ethnicity specific allelic effect reached are indirectly confirmed by the fact that some nonwhite ethnicities with increased COVID-19 mortality exhibit an increased population frequency of the I allele
**C. Socio-cultural**	C1 Kolin et al. 2020: 9	Given the strong associations between deprivation, race, and Covid-19 in this study, our findings may be due, in part, to discriminatory hiring practices within the labor market. Finally, any genetic differences amongst races are highly unlikely to explain our results, as multiple studies have shown that genetic variation within racial groups is greater than between racial groups
	C2 Taylor 2020: 2	From the perspective that not consider social determinants of health on a macroscale, it may seems that race is the underlying factor between differences in disease rates. But examining the context of the data and the racial categories shows that racism (I.e., how an individual categorized into a racial group is treated), and not race, is the cause
**D. Institutional**	D1 Schimmel et al. 2021:2	Race categories were based on the National Hospital Ambulatory Medical Care Survey, using white, black or African American and other
**E. Multileveled**	E1 Ali et al. 2021: 1	Ethnicity is influenced by genetic backgrounds, environmental factors, as well as cultural and behavioral norms. Others have linked ethnicity to socioeconomic status, living standards and occupations (Patel et al. 2020). These factors linked to ethnicity are likely to function interdependently and influence disease outcome
		Odds ratios of dying and being admitted to ICU from COVID-19 if a patient is of South Asian ethnicity. Baseline is Arab ethnicity. ORs are adjusted for age, gender, smoking status and other co-morbidities
	E2 Ali et al. 2021: 2	Results from our cohort, which provided good representation of the population in Kuwait, indicated that South Asians were more likely to develop a severe form of COVID-19 which also corresponded to a higher death rate compared to Arabs
		Our results suggested ethnicity as a possible risk factor for COVID-19 poor prognosis and outcome. The vast majority of South Asians in Kuwait are unskilled labor living in highly populated accommodations. Their living conditions and perhaps occupation can increase their exposure to SARS-CoV2. While this could have a major impact on infection dynamics, it may not explain the results entirely. Local laws grant each resident in Kuwait free access to governmental health care, hence rolling out healthcare access inequalities of such outcome disparity
	E3 Saini et al. 2021: 1	The stark racial disparities related to the coronavirus disease 2019 (COVID-19) pandemic in the United States, wherein minority populations are disproportionately getting infected and succumbing to the disease, is of grave concern. It is critical to understand and address the underlying causes of these disparities that are complex and driven by interacting environmental, social and biological factors. In this article we focus on the African American community and examine how social and environmental determinants of health intersect with biological factors (comorbidities, underlying genetics, host immunity, vitamin D levels, epigenetics) to exacerbate risk for morbidity and mortality
	E4 Saini et al. 2021: 8	Both biological and nonbiological factors seem to contribute to racial disparities in COVID-19

### Results

Most articles did not present any definitions of the racial/ethnic terms, although their uses differed significantly from one to the other (see Table 2). As expected from our search terms, in many articles (71/119 or about 60%), the category of race referred (either implicitly or explicitly) in a reductionist way to some genetic differences between representatives of different populations distinguished according to folk racial classifications (23) or geographical origin (48). Only 14 treated race/ethnicity in a socio-cultural meaning and another 18 assumed that the so-called racial or ethnic differences between populations are influenced by the interaction of a few biological and social factors (we have termed them the multileveled interpretation of racial categories). Finally, in 15 articles, racial categories were applied purely instrumentally due to institutional guidelines (or, for example, researchers’ habits), without any further analysis, explanation, or discussion. Concerning these 15 articles, it is difficult to say unequivocally whether their authors considered these categories (in a genetic, socio-cultural, or multidimensional sense) to be biologically significant from the perspective of genetics and genomics or whether they only took them into account, e.g., for formal requirements.

Moreover, the terms “race” and “racial” were used in 49 of 119 papers (often with the coexistence of the category of ethnicity, especially for articles following the US institutional guidelines), while the term “ethnicity” (without the co-occurring category of race) was used in 70 of them. Interestingly, the category of race appeared most frequently in the socio-cultural and multileveled sense (in a ratio of 19 for “race” to 12 for “ethnicity”). In contrast, when racial divisions were used in the folk-genetic or demogeographic sense, there were most often introduced using the category of ethnicity (in a ratio of 21 for “race” to 51 “for “ethnicity”).

In all of the types of papers we analysed, among those using these categories in a genetic sense, about 33% applied folk racial divisions (23 of 71 papers). Moreover, folk racial classifications largely determine institutional guidelines in the US (Spencer 2018a, 2018b; Winsberg 2019), and thus they were also present in the 16 papers using ethnoracial categories in the institutional sense, raising the percentage of the papers applying it to about 33% of all the analysed articles (39 of 119 papers).

## Limitations

Our case study has several limitations. First, our method neatly encapsulates the discussed phenomena but does not facilitate an evaluation of its scale. Second, our analysis concerned a relatively small number of articles (to keep our corpus manageable to read) and was thematically limited to the literature on a specific topic (Covid-19). Third, we only analysed articles explicitly related to race, ethnicity, genetics, or genomics. Although the choice of database guarantees that the analysed papers were published in reliable biomedical journals (since only such papers are indexed by PubMed on which LitCov is based), we did not examine the disciplines their authors represent. Instead, we wanted to investigate how ethnoracial categories are used and interpreted when they are applied in the context of genetics and genomics (for example, does the mere use of references to genetic and genomic explanations and methods correlate with the biologization of racial categories). Thus, while all these articles are related to genetics and genomics to some degree, not all are written by geneticists. We also did not investigate whether the application and interpretation of racial categories was different in specific areas of biomedical research. This issue requires further study.

## Discussion

We have distinguished two main topics for discussion in this section: (1) the issue of “messiness” in the use of the term “race” and “ethnicity” and (2) the relation between reductionist biases and the biologization of the category of race and ethnicity.

### The “messiness” in the use of the term “race” and “ethnicity”

There are already many voices claiming that the lack of specific, clear, and international guidelines for reporting and using demogeographic categories in research has led to considerable terminological and methodological “mess” that prevents us from, for example, the efficient comparison of different studies or data (López et al. 2017; Huddart et al 2019; Zhang and Finkelstein 2019; Popejoy et al. 2020). Moreover, researchers using racial categories are unsure of their references and report being confused when they need to distinguish between them. In a study conducted on 448 respondents (including 87 non-clinical researchers and 268 genetics professionals), more than two-thirds of participants answered that they were “somewhat” or “not at all confident” in their ability to distinguish between the terms “race, “ethnicity,” and “ancestry” (Popejoy et al. 2020: 71–71).

Nevertheless, most geneticists participating in the study (90%) admitted that racial categories may be an essential source of information, e.g., for contextualizing genetic test results for patients (Popejoy et al. 2020). There are also enormous differences in the way in which racial categories are reported, both between countries and institutions, as well as between individual scientists (López et al. 2017; Huddart et al. 2019; Zhang and Finkelstein 2019). The analysis we conducted supports the above observation (see Table 2).

First, what is termed “race” or “ethnicity” in one article may mean something else in another. This is in line with the opinion that “Although there is some acknowledgement in the biomedical community that racial and ethnic categories are social and not genetic, ideas about race and ethnicity that circulate in biomedicine are contradictory” (Braun 2002: 159). Let us look at some of the quotes cited in Table 2, i.e., C1., E1., B1., D1., and A6. Here, we can clearly see significant differences between the reference in which the terms “race” and “ethnicity” are used and such differences prevent efficient scientific communication. Quote C1. underscores the fact that the position that biologically understood races do not exist, and that so-called “racial differences” in healthcare result rather from strictly socio-cultural reasons. Quote E1. interprets racial categories in such a way that they are dependent on many factors and their interactions with one another, and therefore have a certain biological as well as socio-cultural meaning. There are two possible versions of this multileveled approach. The first assumes that racial categories correspond to differences at the genetic, socio-cultural, or environmental level. The second assumes that there are no human races but only racialised groups and individuals. The process of racialisation in this approach is influenced by factors at various levels—biological, social, etc. (Hochman 2019, 2021b). However, among the texts analysed, we have hardly found any that would share the latter position. Rather, we have found a tendency to biologize race in some texts, despite the early mention of the fact that racial differences in healthcare may be due to various factors (cf. McCoy et al. 2020).

Quote B1. includes the term “ethnic origin” (later in the texts it is also termed “ethnicity” or “ethnic” group”) to refer to demogeographic groups that are meant to designate certain ancestral lines, and thus it is intended to serve as a proxy for looking for genetic polymorphisms etc. Quote D1. points to an institutional understanding of race based on the folk ethnoracial classifications. Finally, quote A6. radically reduces the category of race by identifying it with genetic differences.

We also noticed considerable terminological “mess” when it comes to the separable or interchangeable understanding of terms “race” and “ethnicity”. As the term “ethnicity” has historically usually referred to a person’s cultural identity (e.g., their language, religious beliefs, values, or customs) (Eriksen 2012; Tonkin et al. 2016; Flanagin et al. 2021), when it is employed as a predictor of biological features (e.g., high probability of having certain genes) confusion abounds, especially since many researchers cannot highlight any differences between ethnicity and race. Similarly, the category of ethnicity in some studies referred to cultural identity (in a strictly socio-cultural sense), while in others it meant the country of one’s origin, which was to be a determinant of belonging to a given ancestral group (in a demogeographic, “biological” sense). It is worth adding that these categories are used differently in various countries. For instance, while the category of race in the US (based on the folk racial classifications) is most generally divided into groups such as American Indian and Alaska Indian, Asian, Black or African American, Native Hawaiian or Pacific Islander and White, ethnicity usually refers to Hispanic or Latinx people. In other countries, such as the UK, using the category of race is not recommended, with ethnicity often used as an indicator of a specific nation or ancestry (in the case of the UK, the ethnic classifications used in healthcare are a mixture of folk ethnoracial categories based on phenotypic features like skin colour (a distinction between “white” and “black”), historical contingencies, including the imperial and colonial past (a distinction between “black African” and “black Caribbean”), current political borders (a distinction between “white Irish” and “white British,” with the latter category including the inhabitants of Northern Ireland).

While some researchers divide races from ethnicities, other scientists use the terms “race” and “ethnicity” interchangeably. For example, all of the quotes presented in Table 2 (besides the ones using racial categories in the socio-cultural and multileveled sense) refer to demographic categories (or so-called “genetic groups”) that relate to populations with a great probability that they share a specific set of genes. Thus, this reference is taken by both the terms “race” and “ethnicity”. Sporadically, this interchangeable use of the terms “race” and “ethnicity” can even be found in a single article. For example, in quote A5, the researchers first name three populations: Asian, African American and Caucasian, then they refer to Eastern Asians and “other ethnic groups”, before finally writing about “Asians and other races”.

The greater frequency of use of the term “ethnicity” than “race”, especially understood in the demogeographic sense, is at least to some extent because many of the analysed articles were written by scientists from countries where the category of race is not usually employed, with the term “ethnicity” generally preferred (like in the UK). However, in many cases this was also the case with US researchers, where the terms “race” and “ethnicity” are usually separated. We hypothesize that some researchers may avoid using the controversial category of race and replace it with the category of ethnicity (often used as referring to the ancestral lines, e.g., in the field of pharmacogenetics and pharmacogenomics (Zhang and Finkelstein 2019)) (cf. Byeon et al. 2021). However, considering the fact that the term “ethnicity” for many people refers to cultural phenomena (language, religion, tradition, etc.), treating it as a proxy for designating groups for ancestral or genetic research may lead to the wrong impression that there are biologically understood human races and ethnic groups.

### The relation of methodological reductionism and the biologization of ethnoracial categories

Our findings indicate that while many researchers are aware of the fact that the terms “race” and “ethnicity” rather poorly describe genetic lineage or other biological groups (Popejoy et al. 2020: 77), ethnoracial categories are usually employed in the biological (genetic) sense in most of the analysed articles. Besides factors such as institutional training and guidelines, this may be largely due to the biases related to reductionism.

The most basic “feature” of reductionism that fosters the biologization of social categories is that reductionist methods and explanations usually focus on the internal factors (most often decomposed and cited in isolation) while ignoring or simplifying the environment of the system in the study (cf. Kaiser 2011). It “decomposes a complex system into its parts, analyses them in isolation, and then re-synthesises these parts and the explanation of their behaviour into a composite explanation of some aspect of the behaviour of the system” (Wimsatt 2006: 23; cf. Wimsatt 1974, 1987, 2007; Bechtel and Richardson 1993) and systematically ignores or downplays the context-sensitivity of the research results, as well as the environmental factors influencing it.

The decision into what kind of parts the given system should be decomposed depends on at least three main factors (Kaiser 2011: 14). The first are the current technical and technological possibilities and limitations (e.g., the possibility of observing certain phenomena thanks to the equipment invented for this purpose). Secondly, it depends on the theoretical perspective adopted in a given area of science and the research goals set (Wimsatt 2007: 227). Finally, on the characteristics of the analysed system (Glennan 2002: 344). In the case of genetics and genomics, the most important level of analysis (and the level to which other levels are reduced) is the level of the genes and genome, respectively. While any scientific activity requires the use of simplification, it is important to keep in mind that doing so “transforms the initial problem into one that is easier to analyse and to solve” (Wimsatt 2006: 24). Such a strategy may lead to many potential systematic biases and inaccurate assumptions about the inputs to the system, as well as their effects on its functioning. We believe that one of the effects of these biases is the reduction and biologization of social categories (such as racial categories) used in the genetic and genomic studies as one of the biologically relevant variables.

After Wimsatt (2006), we present several such biases that we find important for our purposes (our reconstruction of Wimsatt’s proposal is narrowed down to the issues that we recognise as relevant from the perspective of this article).

1. The assumption that “all descriptions and processes are to be referred to entities at a given level, which are particularly robust, salient, or provide an apparently combinatorial basis for the construction of other entities and properties” (Wimsatt 2006: 25), which is the “ontological equivalent of assuming that there is a single cause for a phenomenon, or single level at which causation can act” (Wimsatt 2006: 25). Moreover, according to Wimsatt, such a perspective can lead to what he calls as a “project of ‘level completion’” (cf. The Human Genome Project) i.e., the idea that the aim of a certain investigation can be narrowed to a single level followed by the conviction that a complete description of entities or phenomena at that level will be sufficient to solve the problems that occur on this and other levels (Wimsatt 2006: 25). Trying to describe and explain so-called “racial or ethnic differences” in health by sole reference to genetic polymorphism etc. amounts to misplacing a research problem. That is, if we consider that genetic research is meant to explain the differences between representatives of certain social categories, then we are either looking in the wrong place or assuming a total reduction of social categories to biological categories (cf. A5, A6, B4, B5, B6). In both cases, it leads to the biologization of racial categories and especially if one assumes that treating racial categories as a proxy allowing for the identification of genetically similar populations is not scientifically justified.
2. The tendency to simplify the environment before simplifying the analysed system, which may lead to the impression that the environment is somewhat homogenous, constant, and regular and, in effect, causes the complete bypassing or downplaying of higher systems (Wimsatt 2006: 25–26). This tendency is also associated with the disposition to omit the monitoring of environmental variables and thus failing to detect interactional or larger scale patterns as well as keeping environmental variables constant while missing dependencies of system variables on them, i.e., on the environmental variables (Wimsatt 2006: 27). Ignoring the environmental factors influencing the differences in health between representatives of different populations is, at the current state of knowledge regarding epigenetics and developmental (phenotypic) plasticity, for example, a serious mistake. Genetic differences between populations certainly do exist and should be carefully studied (although they do not correspond to ethnoracial categories). Yet overlooking environmental variables and assuming that genetic differences are the main causes of differences in health between representatives of certain “races” or “ethnicities” lead to a failure to detect the real sources of these differences as well as to the erroneous notion that they are indeed rooted in biological differences (and therefore, once again, to the biologization of ethnoracial categories) (cf. Block 1995). In other words, when the influence of external factors on the situation is ignored, then it may be automatically assumed that differences in the health of representatives of different “races” and “ethnicities” are due to the genetic differences between them. In this way, the notion that there is indeed some biological (genetic) difference between representatives of racialised groups is strengthened even though, after all, belonging to the racial category is a very unprecise proxy for determining the genetic heritage of a given person. Such a research perspective seems close to some of the researchers whose articles we analysed. To check this assumption, let us return to Table 2, and look more closely at quotes A3., A7., B2., B3. and B5. They all indicate that specific genetic or biological differences, which appear to be distributed differently across races or ethnic groups, are considered to be the most significant in explaining racial differences in the contraction and mortality from Covid-19. And while there are researchers who see these differences as being caused by complex, systemic, and environmental factors (cf. C1., C2., E1, E2., E3. and E4.), the tendency to simplify this problem, one clearly discernible in many of the articles, continues to be of concern.
3. Observing or modelling only regularities and things that are common to all analysed cases while ignoring individual circumstances (Wimsatt 2006: 26). Focusing on regularities when analysing genetic “racial differences” in health while ignoring individual circumstances can lead to the erroneous impression that all representatives of the studied populations have the same, common, and universal genetic features (which other people do not have), and thus to the essentialisation of human races and ethnic groups. For example, in quotes A2. and B6., the emphasis has been placed on the fact that the representatives of the distinguished racial groups share a common feature, i.e., the possession of specific genes. However, the use of such imprecise categories as “nonwhite ethnicities” in this case may mislead and reinforce the belief that there is some biologically distinguished “white” and “nonwhite” “race” or “ethnicity”. Similarly, it is questionable that one can maintain that African Americans constitute a genetically homogeneous “ethnic group”. Emphasizing the common genetic features of representatives of the distinguished groups (features that representative of other “races” or “ethnicities” do not have) may lead to the essentialisation of racial categories.
4. A belief that a system can be “exhaustively described and explained from a given perspective because it has been very successfully and powerfully so described” (Wimsatt 2006: 28). For example, it may be a misconception that all of the problems related to the processes of the aging of the organism can be solved thanks to genetic or molecular research because these disciplines have been successful in describing these processes (while, in reality, aging also depends on environmental factors). Such a “perceptual focus” can inflate the convictions of researchers about what properties and processes can be efficiently analysed in their research field (cf. B3, B4, B5, B6).
5. Finally, the inclination to become “sufficiently bound to a specific tool” (Wimsatt 2006: 28), i.e., using the same research methods and tools over and over again and just looking for new problems that they can solve rather than the opposite (selecting appropriate research methods and tools for solving a specific problem). The belief that the tools of genetics and genomics can fully describe all phenomena related to human health (or development, behaviour, traits etc.) can lead to the essentialization of racial categories. This is because it leads to the formulation of scientific projects aimed at describing and explaining compounded, multi-level phenomena with tools that, by definition, downplay the context and complexity of the analysed situation (cf. A2, A3, B3, B4, B5, B6). Thus, together with previous bias (4), it can only strengthen the effects of all the reductionist tendencies which geneticists are exposed to, and which have been described in this section.

## Conclusions: towards an ethics of categorization for medical research and treatment

In this article, we have reconstructed and analysed what researchers refer to when they use ethnoracial categories with reference to genetics and genomics when publishing in biomedical journals. Although we noticed a large variety of positions, the prevailing tendency among researchers was to biologize these categories. We have also noticed a tendency to replace the category of race with that of “ethnicity” as a proxy for a genetic lineage, which agrees with recent studies by Byeon et al. (2021). We approach this phenomenon critically—we are of the opinion that instead of contributing to minimalising or solving the problem of biologizing race, it may lead to the biologisation of the category of ethnicity.

Finally, when it comes to the biological interpretation of ethnoracial categories with reference to genetics and genomics, we believe that to a point (apart from factors such as education, conventions prevailing in a given scientific environment, institutional requirements, etc.), it may be due to some reductionist biases implicitly contained in genetic methodologies and explanations. By focusing on genes and genetic differences, researchers are geared to a series of epistemic risks that may lead to the biologization of the categories they employ. Knowing these risks can increase one’s methodological awareness and contribute to more reliable research practices. The manner in which participants in biomedical research are categorised and selected actively co-shapes the subsequent research results. These categories are not “discovered” by scientists; they are not natural kinds but rather constructs built on the process of idealization of certain populations to obtain specific scientific, political, economic, or cultural goals. Although this problem is clearly discernible in the case of races, it applies to other population categories used in biomedical research. Therefore, we believe that it would be desirable to define what values are behind each scientific decision in this regard: something that has not yet been properly investigated transparently (see the discussion concerning risk stratification in John (2013)).

In particular, we believe that current institutional requirements in the US (in contrast to the majority of EU countries) may reinforce the assumption that ethnoracial categories are relevant because the institutions that regulate research require them to be used. Moreover, US regulatory standards are disseminated in other parts of the world thanks to the harmonization of drug testing and regulation. For example, more EU than US pharmaceutical product labels contain statements about racial and ethnic differences and there is evidence that reporting of ethnoracial demographics in the labels of all novel drugs in the EU may be driven, at least in part, by statements on US labels (Mulinari et al., 2021). Researchers all over the world are thus constantly encouraged to look for information to corroborate hypotheses about the relation between races/ethnicities and disease or treatment options. Sometimes, as with many other variables, they do find some correlations. However, since what the ethnoracial categories used in the studies refer to is ambiguous in many cases, it is also difficult to reliably interpret such research results and identify the causes of the observed correlations (if there are such). In this sense, even the use of ethnoracial categories interpreted in a socio-cultural way raises doubts. People have a variety of social identities and enter various relations of power and discrimination that affect their health (cf. Krieger 2021). These intersectional relations cannot be reduced to a single proxy such as race and ethnicity. For instance, although people identifying themselves as belonging to ethnoracial minorities are constantly exposed to a number of social exclusions etc., scientists shouldn’t equate racial affiliations with socioeconomic status e.g.., due to the fact that not all representatives of ethnoracial minorities are in similar economic situations. So, what do researchers using socially interpreted ethnoracial categories in biomedical research refer to? What aspects of the impact of systemic racism on health are they studying? When such questions remain unanswered, studies referring to these categories have very limited (if any) explanatory value and contribute to the reinforcement of racial stereotypes.

Our proposal is not to remove all uses of ethnoracial classifications in research but rather a subtler two-pronged approach. Firstly, we should require regulators and researchers alike to justify why they want to use a certain form of a given category (e.g., self-declared ethnoracial affiliations) as a variable and a proxy in their research. Secondly, we should encourage them to report socioeconomic status and deprivation using disadvantage indices or other suitable measures. This would help to convey and reinforce the fact that racism, rather than race, is what drives major disparities in health.

## Supplementary Information

Below is the link to the electronic supplementary material.

Supplementary file1 (DOCX 37 KB)

## Footnotes

1 The development of genetics is closely related to the development of reductionist thinking (mainly reductionist methodology) within the scope of biomedical research and other fields of life sciences (Sarkar 1998; Brigandt and Love 2008; Griffiths and Stotz 2013; Rose and Rutledge 2019). Although other perspectives are being developed in the area of genetics and genomics (cf. DiFrisco and Jaeger 2020), we recognize that methodological reductionism continues to be the dominant paradigm in those disciplines.

2 The term “messy” is commonly used by philosophers of biology, e.g., “It has seemed to many philosophers that chemistry was a bastion of monism—roughly, the view that there is just one good way of dividing things up. On the other hand, things get messy in the biological world” (Slater 2009).

3 https://www.ncbi.nlm.nih.gov/research/coronavirus/ Access: September 3, 2021.

4 Journal selection criteria can be found here: https://www.ncbi.nlm.nih.gov/pmc/pub/journalselect/ and https://clarivate.com/webofsciencegroup/solutions/editorial-selection-process/ Access: September 3, 2021.

5 For instance, thanks to the reference to an article directly criticizing the biological significance of racial categories (Lewis 2002), we could more confidently conclude that Taylor (2020) recognizes these categories in a more socio-cultural manner.
