# Supplementary material for: Reductionist methodology and the ambiguity of the categories of race and ethnicity in biomedical research: an exploratory study of recent evidence
Source: Med Health Care Philos. 2022 Nov 9;26(1):55–68. doi: 10.1007/s11019-022-10122-y (PMC9646278; doi:10.1007/s11019-022-10122-y)
Supplement: Supplementary file 1 — Supplementary file1 (DOCX 37 KB) [file 11019_2022_10122_MOESM1_ESM.docx]

**Supplement to the bibliography**

A corpus of papers analysed in the paper: The ambiguity of the categories of race and ethnicity in biomedical research: the case of Covid-19.

1. Abbasi, A. Z., Kiyani, D. A., Hamid, S. M., Saalim, M., Fahim, A., & Jalal, N. (2021). Spiking dependence of SARS‐CoV‐2 pathogenicity on TMPRSS2. *Journal of Medical Virology*, *93*(7), 4205-4218.
2. AbdelMassih, A. F., Fouda, R., Kamel, A., Mishriky, F., Ismail, H. A., El Qadi, L., ... & Ye, J. (2020). Single cell sequencing unraveling genetic basis of severe COVID19 in obesity. *Obesity Medicine*, *20*, 100303.
3. Abdelzaher, H., Saleh, B. M., Ismail, H. A., Hafiz, M., Gabal, M. A., Mahmoud, M., ... & Abdelnaser, A. (2020). COVID-19 genetic and environmental risk factors: A look at the evidence. *Frontiers in pharmacology*, *11*, 1528.
4. Ahmed, M. H. (2020). Black and minority ethnic (BAME) alliance against COVID-19: one step forward. *Journal of racial and ethnic health disparities*, *7*(5), 822-828.
5. Airhihenbuwa, C. O., Tseng, T. S., Sutton, V. D., & Price, L. (2021). Non–Peer Reviewed: Global Perspectives on Improving Chronic Disease Prevention and Management in Diverse Settings. *Preventing Chronic Disease*, *18*.
6. Al-Dayan, N., Venugopal, D., & Dhanasekaran, S. (2020). Footprint of the COVID-19 Pandemic in India: A Study of Immune Landscape and Other Factors Shielding Mortality. *Analytical Cellular Pathology*, *2020*.
7. Al-Jaf, S. M., Niranji, S. S., Ali, H. N., & Mohammed, O. A. (2021). Association of Apolipoprotein e polymorphism with SARS-CoV-2 infection. *Infection, Genetics and Evolution*, *95*, 105043.
8. Ali, H., Alshukry, A., Marafie, S. K., AlRukhayes, M., Ali, Y., Abbas, M. B., ... & Al-Mulla, F. (2021). Outcomes of COVID-19: Disparities by ethnicity. *Infection, Genetics and Evolution*, *87*, 104639.
9. Alsaied, T., & Ashfaq, A. (2020). From Other Journals: A Review of Recent Articles in Pediatric Cardiology. *Pediatric cardiology*, 1-6.
10. Mallavarpu Ambrose, J., Priya Veeraraghavan, V., Kullappan, M., Chellapandiyan, P., Krishna Mohan, S., & Manivel, V. A. (2021). Comparison of Immunological Profiles of SARS-CoV-2 Variants in the COVID-19 Pandemic Trends: An Immunoinformatics Approach. *Antibiotics*, *10*(5), 535.
11. Aviv, A. (2021). Short telomeres and severe COVID-19: The connection conundrum. *EBioMedicine*, *70*.
12. Bailey, L. C., Razzaghi, H., Burrows, E. K., Bunnell, H. T., Camacho, P. E., Christakis, D. A., ... & Forrest, C. B. (2021). Assessment of 135 794 pediatric patients tested for severe acute respiratory syndrome coronavirus 2 across the United States. *JAMA pediatrics*, *175*(2), 176-184.
13. Bakhshandeh, B., Jahanafrooz, Z., Abbasi, A., Goli, M. B., Sadeghi, M., Mottaqi, M. S., & Zamani, M. (2021). Mutations in SARS-CoV-2; Consequences in structure, function, and pathogenicity of the virus. *Microbial Pathogenesis*, 104831.
14. Bao, R., Hernandez, K., Huang, L., & Luke, J. J. (2020). ACE2 and TMPRSS2 expression by clinical, HLA, immune, and microbial correlates across 34 human cancers and matched normal tissues: implications for SARS-CoV-2 COVID-19. *Journal for immunotherapy of cancer*, *8*(2).
15. von Bartheld, C. S., Hagen, M. M., & Butowt, R. (2020). Prevalence of chemosensory dysfunction in COVID-19 patients: a systematic review and meta-analysis reveals significant ethnic differences. *ACS chemical neuroscience*, *11*(19), 2944-2961.
16. Barash, A., Machluf, Y., Ariel, I., & Dekel, Y. (2020). The pursuit of COVID-19 biomarkers: putting the spotlight on ACE2 and TMPRSS2 regulatory sequences. *Frontiers in medicine*, *7*.
17. Baumer, T., Phillips, E., Dhadda, A., & Szakmany, T. (2020). Epidemiology of the first wave of COVID-19 ICU admissions in South Wales—the interplay between ethnicity and deprivation. *Frontiers in Medicine*, *7*, 650.
18. Bhattacharyya, P. (2020). Multisystem Inflammatory Syndrome of Children Related to SARS-CoV-2: A Novel Experience in Children with a Novel Virus. *Indian Journal of Critical Care Medicine: Peer-reviewed, Official Publication of Indian Society of Critical Care Medicine*, *24*(11), 1010.
19. Biswas, N. K., & Majumder, P. P. (2020). Analysis of RNA sequences of 3636 SARS-CoV-2 collected from 55 countries reveals selective sweep of one virus type. *The Indian journal of medical research*, *151*(5), 450.
20. Bunyavanich, S., Grant, C., & Vicencio, A. (2020). Racial/ethnic variation in nasal gene expression of transmembrane serine protease 2 (TMPRSS2). *Jama*, *324*(15), 1567-1568
21. Cardenas, A., Rifas-Shiman, S. L., Sordillo, J. E., DeMeo, D. L., Baccarelli, A. A., Hivert, M. F., ... & Oken, E. (2021). DNA methylation architecture of the ACE2 gene in nasal cells of children. *Scientific reports*, *11*(1), 1-9.
22. Carethers, J. M. (2021). Insights into disparities observed with COVID‐19. *Journal of internal medicine*, *289*(4), 463-473.
23. Challen, R., Brooks-Pollock, E., Read, J. M., Dyson, L., Tsaneva-Atanasova, K., & Danon, L. (2021). Risk of mortality in patients infected with SARS-CoV-2 variant of concern 202012/1: matched cohort study. *bmj*, *372*.
24. Chamie, G., Marquez, C., Crawford, E., Peng, J., Petersen, M., Schwab, D., ... & Havlir, D. V. (2021). Community transmission of severe acute respiratory syndrome coronavirus 2 disproportionately affects the Latinx population during shelter-in-place in San Francisco. *Clinical Infectious Diseases*, *73*(Supplement_2), S127-S135.
25. Chang, L., Yang, H. W., Lin, T. Y., & Yang, K. D. (2021). Perspective of Immunopathogenesis and Immunotherapies for Kawasaki Disease. *Frontiers in Pediatrics*, 719.
26. Chen, H. H., Shaw, D. M., Petty, L. E., Graff, M., Bohlender, R. J., Polikowsky, H. G., ... & Below, J. E. (2021). Host genetic effects in pneumonia. *The American Journal of Human Genetics*, *108*(1), 194-201.
27. Chen, J., Jiang, Q., Xia, X., Liu, K., Yu, Z., Tao, W., ... & Han, J. D. J. (2020). Individual variation of the SARS‐CoV‐2 receptor ACE2 gene expression and regulation. *Aging cell*, *19*(7), e13168.
28. Davies, N. G., Jarvis, C. I., Edmunds, W. J., Jewell, N. P., Diaz-Ordaz, K., & Keogh, R. H. (2021). Increased mortality in community-tested cases of SARS-CoV-2 lineage B. 1.1. 7. *Nature*, *593*(7858), 270-274.
29. Diwan, S. (2020). Covid-19 pandemic: Implications on interventional pain practice—a narrative review. *Pain Physician*, *23*, S311-S318.
30. Dutta, A. K., & Goswami, K. (2021). Host genomics of COVID-19: Evidence point towards Alpha 1 antitrypsin deficiency as a putative risk factor for higher mortality rate. *Medical hypotheses*, *147*, 110485.
31. Ebrahimi, S., Ghasemi-Basir, H. R., Majzoobi, M. M., Rasouli-Saravani, A., Hajilooi, M., & Solgi, G. (2021). HLA-DRB1* 04 may predict the severity of disease in a group of Iranian COVID-19 patients. *Human immunology*.
32. Elsayed, Y., & Khan, N. A. (2020). Immunity-boosting spices and the novel coronavirus. *ACS chemical neuroscience*, *11*(12), 1696-1698.
33. Emeny, R. T., Carpenter, D. O., & Lawrence, D. A. (2021). Health disparities: Intracellular consequences of social determinants of health. *Toxicology and Applied Pharmacology*, 115444.
34. Fujikura, K., & Uesaka, K. (2021). Genetic variations in the human severe acute respiratory syndrome coronavirus receptor ACE2 and serine protease TMPRSS2. *Journal of Clinical Pathology*, *74*(5), 307-313.
35. Gelaye, B., Foster, S., Bhasin, M., Tawakol, A., & Fricchione, G. (2020). SARS-CoV-2 morbidity and mortality in racial/ethnic minority populations: A window into the stress related inflammatory basis of health disparities. *Brain, behavior, & immunity-health*, 100158.
36. Gemmati, D., & Tisato, V. (2020). Genetic hypothesis and pharmacogenetics side of renin-angiotensin-system in COVID-19. *Genes*, *11*(9), 1044.
37. Getachew, B., & Tizabi, Y. (2021). Vitamin D and COVID‐19: Role of ACE2, Age, Gender and Ethnicity. *Journal of medical virology*.
38. Ghafouri-Fard, S., Noroozi, R., Vafaee, R., Branicki, W., Poṡpiech, E., Pyrc, K., ... & Sanak, M. (2020). Effects of host genetic variations on response to, susceptibility and severity of respiratory infections. *Biomedicine & Pharmacotherapy*, *128*, 110296.
39. Gkouskou, K., Vasilogiannakopoulou, T., Andreakos, E., Davanos, N., Gazouli, M., Sanoudou, D., & Eliopoulos, A. G. (2021). COVID-19 enters the expanding network of apolipoprotein E4-related pathologies. *Redox biology*, 101938.
40. Gorecka, M., McCann, G. P., Berry, C., Ferreira, V. M., Moon, J. C., Miller, C. A., ... & Greenwood, J. P. (2021). Demographic, multi-morbidity and genetic impact on myocardial involvement and its recovery from COVID-19: protocol design of COVID-HEART—a UK, multicentre, observational study. *Journal of Cardiovascular Magnetic Resonance*, *23*(1), 1-13
41. Göreke, V., Sarı, V., & Kockanat, S. (2021). A novel classifier architecture based on deep neural network for COVID-19 detection using laboratory findings. *Applied Soft Computing*, *106*, 107329.
42. Griffin, G., Hewison, M., Hopkin, J., Kenny, R., Quinton, R., Rhodes, J., ... & Thickett, D. (2020). Vitamin D and COVID-19: evidence and recommendations for supplementation. *Royal Society open science*, *7*(12), 201912.
43. Gupta, R., & Misra, A. (2020). COVID19 in South Asians/Asian Indians: heterogeneity of data and implications for pathophysiology and research. *diabetes research and clinical practice*, *165*, 108267.
44. Hachim, M. Y., Al Heialy, S., Senok, A., Hamid, Q., & Alsheikh-Ali, A. (2020). Molecular basis of cardiac and vascular injuries associated with COVID-19. *Frontiers in cardiovascular medicine*, *7*.
45. Hanan, N., Doud, R. L., Park, I. W., Jones, H. P., & Mathew, S. O. (2021). The Many Faces of Innate Immunity in SARS-CoV-2 Infection. *Vaccines*, *9*(6), 596.
46. Hisaka, A., Yoshioka, H., Hatakeyama, H., Sato, H., Onouchi, Y., & Anzai, N. (2020). Global comparison of changes in the number of test-positive cases and deaths by coronavirus infection (COVID-19) in the world. *Journal of clinical medicine*, *9*(6), 1904.
47. Hu, J., Li, C., Wang, S., Li, T., & Zhang, H. (2021). Genetic variants are identified to increase risk of COVID-19 related mortality from UK Biobank data. *Human genomics*, *15*(1), 1-10.
48. Hubacek, J. A., Dusek, L., Majek, O., Adamek, V., Cervinkova, T., Dlouha, D., & Adamkova, V. (2021). ACE I/D polymorphism in Czech first-wave SARS-CoV-2-positive survivors. *Clinica Chimica Acta*, *519*, 206-209.
49. Ishii, T., Kushimoto, S., Katori, Y., Kure, S., Igarashi, K., Fujita, M., ... & Akaishi, T. (2021). Predictors of SARS-CoV-2 positivity based on RT-PCR swab tests at a drive-through outpatient clinic for COVID-19 screening in Japan. *The Tohoku Journal of Experimental Medicine*, *253*(2), 101-108.
50. Jain, S. K., Parsanathan, R., Levine, S. N., Bocchini, J. A., Holick, M. F., & Vanchiere, J. A. (2020). The potential link between inherited G6PD deficiency, oxidative stress, and vitamin D deficiency and the racial inequities in mortality associated with COVID-19. *Free Radical Biology and Medicine*, *161*, 84-91.
51. Kantri, A., Ziati, J., Khalis, M., Haoudar, A., El Aidaoui, K., Daoudi, Y., ... & El Kettani, C. (2021). Hematological and biochemical abnormalities associated with severe forms of COVID-19: A retrospective single-center study from Morocco. *Plos one*, *16*(2), e0246295.
52. Kaur, U., Chakrabarti, S. S., & Patel, T. K. (2021). Renin–angiotensin–aldosterone system blockers and region-specific variations in COVID-19 outcomes: findings from a systematic review and meta-analysis. *Therapeutic advances in drug safety*, *12*, 20420986211011345.
53. Khayat, A. S., De Assumpção, P. P., Meireles Khayat, B. C., Thomaz Araújo, T. M., Batista-Gomes, J. A., Imbiriba, L. C., ... & Dos Santos, S. E. B. (2020). ACE2 polymorphisms as potential players in COVID-19 outcome. *PloS one*, *15*(12), e0243887.
54. Kim, Y. C., & Jeong, B. H. (2021). Strong correlation between the case fatality rate of COVID-19 and the rs6598045 single nucleotide polymorphism (SNP) of the interferon-induced transmembrane protein 3 (IFITM3) gene at the population-level. *Genes*, *12*(1), 42.
55. Kolin, D. A., Kulm, S., Christos, P. J., & Elemento, O. (2020). Clinical, regional, and genetic characteristics of Covid-19 patients from UK Biobank. *Plos one*, *15*(11), e0241264.
56. Kwan, P. K. W., Cross, G. B., Naftalin, C. M., Ahidjo, B. A., Mok, C. K., Fanusi, F., ... & Paton, N. I. (2021). A blood RNA transcriptome signature for COVID-19. *BMC medical genomics*, *14*(1), 1-8.
57. Langton, D. J., Bourke, S. C., Lie, B. A., Reiff, G., Natu, S., Darlay, R., ... & Echevarria, C. (2021). The influence of HLA genotype on the severity of COVID‐19 infection. *HLA*.
58. Lee, I. H., Lee, J. W., & Kong, S. W. (2020). A survey of genetic variants in SARS-CoV-2 interacting domains of ACE2, TMPRSS2 and TLR3/7/8 across populations. *Infection, Genetics and Evolution*, *85*, 104507.
59. Lesho, E., Reno, L., Newhart, D., Clifford, R., Vasylyeva, O., Hanna, J., ... & Walsh, E. (2020). Temporal, Spatial, and Epidemiologic Relationships of Severe Acute Respiratory Syndrome Coronavirus 2 (SARS-CoV-2) Gene Cycle Thresholds: A Pragmatic Ambi-Directional Observation. *Clinical Infectious Diseases*.
60. Li, M. Y., Li, L., Zhang, Y., & Wang, X. S. (2020). Expression of the SARS-CoV-2 cell receptor gene ACE2 in a wide variety of human tissues. *Infectious diseases of poverty*, *9*(1), 1-7.
61. Li, Q., Cao, Z., & Rahman, P. (2020). Genetic variability of human angiotensin‐converting enzyme 2 (hACE2) among various ethnic populations. *Molecular genetics & genomic medicine*, *8*(8), e1344.
62. Loney, T., Khansaheb, H., Ramaswamy, S., Harilal, D., Deesi, Z. O., Varghese, R. M., ... & Abou Tayoun, A. (2021). Genotype‐phenotype correlation identified a novel SARS‐CoV‐2 variant possibly linked to severe disease. *Transboundary and emerging diseases*.
63. Long, S. W., Olsen, R. J., Christensen, P. A., Bernard, D. W., Davis, J. J., Shukla, M., ... & Musser, J. M. (2020). Molecular architecture of early dissemination and massive second wave of the SARS-CoV-2 virus in a major metropolitan area. *MBio*, *11*(6), e02707-20.
64. Loo, K. Y., Letchumanan, V., Ser, H. L., Teoh, S. L., Law, J. W. F., Tan, L. T. H., ... & Lee, L. H. (2021). COVID-19: Insights into potential vaccines. *Microorganisms*, *9*(3), 605.
65. Luo, R., Delaunay‐Moisan, A., Timmis, K., & Danchin, A. (2021). SARS‐CoV‐2 biology and variants: anticipation of viral evolution and what needs to be done.
66. Maison, D. P., Ching, L. L., Shikuma, C. M., & Nerurkar, V. R. (2021). Genetic Characteristics and Phylogeny of 969-bp S Gene Sequence of SARS-CoV-2 from Hawai ‘i Reveals the Worldwide Emerging P681H Mutation. *Hawai'i journal of health & social welfare*, *80*(3), 52.
67. Martín Giménez, V. M., Ferder, L., Inserra, F., García, J., & Manucha, W. (2020). Differences in RAAS/vitamin D linked to genetics and socioeconomic factors could explain the higher mortality rate in African Americans with COVID-19. *Therapeutic Advances in Cardiovascular Disease*, *14*, 1753944720977715.
68. Martynowicz, H., Jodkowska, A., Poręba, R., Mazur, G., & Więckiewicz, M. (2021). Demographic, clinical, laboratory, and genetic risk factors associated with COVID-19 severity in adults: A narrative review. *Dental and Medical Problems*, *58*(1), 115-121.
69. McCoy, J., Wambier, C. G., Vano‐Galvan, S., Shapiro, J., Sinclair, R., Ramos, P. M., ... & Goren, A. (2020). Racial variations in COVID‐19 deaths may be due to androgen receptor genetic variants associated with prostate cancer and androgenetic alopecia. Are anti‐androgens a potential treatment for COVID‐19?. *Journal of cosmetic dermatology*.
70. Mehta, P., Machado, P. M., & Gupta, L. (2021). Understanding and managing anti-MDA 5 dermatomyositis, including potential COVID-19 mimicry. *Rheumatology International*, 1-16.
71. Mehrbod, P., Eybpoosh, S., Farahmand, B., Fotouhi, F., & Alishahi, M. K. (2021). Association of the host genetic factors, hypercholesterolemia and diabetes with mild influenza in an Iranian population. *Virology journal*, *18*(1), 1-11.
72. Mohammed, F. S., Farooqi, Y. N., & Mohammed, S. (2021). The Interferon-Induced Transmembrane Protein 3-rs12252 Allele May Predict COVID-19 Severity Among Ethnic Minorities. *Frontiers in genetics*, *12*.
73. Naemi, F. M., Al‐adwani, S., Al‐khatabi, H., & Al‐nazawi, A. (2021). Association between the HLA genotype and the severity of COVID‐19 infection among South Asians. *Journal of Medical Virology*.
74. Newman, L. A., Winn, R. A., & Carethers, J. M. (2021). Similarities in risk for COVID-19 and cancer disparities. *Clinical Cancer Research*, *27*(1), 24-27.
75. Newman, L., Fejerman, L., Pal, T., Mema, E., McGinty, G., Cheng, A., ... & Hunt, K. (2021). Breast Cancer Disparities Through the Lens of the COVID-19 Pandemic. *Current breast cancer reports*, 1-3.
76. Nikoloudis, D., Kountouras, D., & Hiona, A. (2020). The frequency of combined IFITM3 haplotype involving the reference alleles of both rs12252 and rs34481144 is in line with COVID-19 standardized mortality ratio of ethnic groups in England. *PeerJ*, *8*, e10402.
77. Novelli, A., Biancolella, M., Borgiani, P., Cocciadiferro, D., Colona, V. L., D’Apice, M. R., ... & Novelli, G. (2020). Analysis of ACE2 genetic variants in 131 Italian SARS-CoV-2-positive patients. *Human genomics*, *14*(1), 1-6.
78. Nyberg, T., Twohig, K. A., Harris, R. J., Seaman, S. R., Flannagan, J., Allen, H., ... & Presanis, A. M. (2021). Risk of hospital admission for patients with SARS-CoV-2 variant B. 1.1. 7: cohort analysis. *bmj*, *373*.
79. Obajuluwa, A. O., Okiki, P. A., Obajuluwa, T. M., & Afolabi, O. B. (2020). In-silico nucleotide and protein analyses of S-gene region in selected zoonotic coronaviruses reveal conserved domains and evolutionary emergence with trajectory course of viral entry from SARS-CoV-2 genomic data. *The Pan African Medical Journal*, *37*.
80. Oyarzun, P., Kashyap, M., Fica, V., Salas-Burgos, A., Gonzalez-Galarza, F. F., McCabe, A., ... & Kobe, B. (2021). A proteome-wide immunoinformatics tool to accelerate T-cell epitope discovery and vaccine design in the context of emerging infectious diseases: an ethnicity-oriented approach. *Frontiers in immunology*, *12*.
81. Pabalan, N., Tharabenjasin, P., Suntornsaratoon, P., Jarjanazi, H., & Muanprasat, C. (2021). Ethnic and age-specific acute lung injury/acute respiratory distress syndrome risk associated with angiotensin-converting enzyme insertion/deletion polymorphisms, implications for COVID-19: A meta-analysis. *Infection, Genetics and Evolution*, *88*, 104682.
82. Parsa, S., Mogharab, V., Ebrahimi, M., Ahmadi, S. R., Shahi, B., Mehramiz, N. J., ... & Hatami, N. (2021). COVID-19 as a worldwide selective event and bitter taste receptor polymorphisms: An ecological correlational study. *International journal of biological macromolecules*, *177*, 204-210.
83. Pathak, G. A., Singh, K., Miller-Fleming, T. W., Wendt, F. R., Ehsan, N., Hou, K., ... & Mancuso, N. (2021). Integrative genomic analyses identify susceptibility genes underlying COVID-19 hospitalization. *Nature communications*, *12*(1), 1-11.
84. Pathangey, G., Fadadu, P. P., Hospodar, A. R., & Abbas, A. E. (2021). Angiotensin-converting enzyme 2 and COVID-19: patients, comorbidities, and therapies. *American Journal of Physiology-Lung Cellular and Molecular Physiology*, *320*(3), L301-L330.
85. Pereira, N. L., Ahmad, F., Cummins, N. W., Byku, M., Morris, A. A., Owens, A., ... & Cresci, S. (2020, December). COVID-19: understanding inter-individual variability and implications for precision medicine. In *Mayo Clinic Proceedings*. Elsevier.
86. Peters, M. C., Sajuthi, S., Deford, P., Christenson, S., Rios, C. L., Montgomery, M. T., ... & Fahy, J. V. (2020). COVID-19–related genes in sputum cells in asthma. Relationship to demographic features and corticosteroids. *American journal of respiratory and critical care medicine*, *202*(1), 83-90.
87. Phillips, N., Park, I. W., Robinson, J. R., & Jones, H. P. (2020). The perfect storm: COVID-19 health disparities in US Blacks. *Journal of racial and ethnic health disparities*, 1-8.
88. Piva, F., Sabanovic, B., Cecati, M., & Giulietti, M. (2021). Expression and co-expression analyses of TMPRSS2, a key element in COVID-19. *European Journal of Clinical Microbiology & Infectious Diseases*, *40*(2), 451-455.
89. Pollard, C. A., Morran, M. P., & Nestor-Kalinoski, A. L. (2020). The COVID-19 pandemic: a global health crisis. *Physiological Genomics*, *52*(11), 549-557.
90. Ponti, G., Pastorino, L., Manfredini, M., Ozben, T., Oliva, G., Kaleci, S., ... & Tomasi, A. (2021). COVID‐19 spreading across world correlates with C677T allele of the methylenetetrahydrofolate reductase (MTHFR) gene prevalence. *Journal of Clinical Laboratory Analysis*, e23798.
91. Rahman, M. S., Hoque, M. N., Islam, M. R., Akter, S., Alam, A. R. U., Siddique, M. A., ... & Hossain, M. A. (2020). Epitope-based chimeric peptide vaccine design against S, M and E proteins of SARS-CoV-2, the etiologic agent of COVID-19 pandemic: an in silico approach. *PeerJ*, *8*, e9572.
92. Raisi-Estabragh, Z., McCracken, C., Bethell, M. S., Cooper, J., Cooper, C., Caulfield, M. J., ... & Petersen, S. E. (2020). Greater risk of severe COVID-19 in Black, Asian and Minority Ethnic populations is not explained by cardiometabolic, socioeconomic or behavioural factors, or by 25 (OH)-vitamin D status: study of 1326 cases from the UK Biobank. *Journal of Public Health*, *42*(3), 451-460.
93. Ravaioli, S., Tebaldi, M., Fonzi, E., Angeli, D., Mazza, M., Nicolini, F., ... & Bravaccini, S. (2020). ACE2 and TMPRSS2 potential involvement in genetic susceptibility to SARS-COV-2 in cancer patients. *Cell transplantation*, *29*, 0963689720968749.
94. Reid, J. A., & Mabhala, M. A. (2021). Ethnic and minority group differences in engagement with COVID-19 vaccination programmes–at Pandemic Pace; when vaccine confidence in mass rollout meets local vaccine hesitancy. *Israel Journal of Health Policy Research*, *10*(1), 1-9.
95. Rhodes, J. M., Subramanian, S., Laird, E., Griffin, G., & Kenny, R. A. (2021). Perspective: Vitamin D deficiency and COVID‐19 severity–plausibly linked by latitude, ethnicity, impacts on cytokines, ACE2 and thrombosis. *Journal of internal medicine*, *289*(1), 97-115.
96. Rutledge, S. M., Schiano, T. D., Florman, S., & Im, G. Y. (2021). COVID‐19 Aftershocks on Alcohol‐associated Liver Disease: An Early Cross‐Sectional Report from the US Epicenter. *Hepatology communications*.
97. Sahana, S., Sivadas, A., Mangla, M., Jain, A., Bhoyar, R. C., Pandhare, K., ... & Scaria, V. (2021). Pharmacogenomic landscape of COVID-19 therapies from Indian population genomes. *Pharmacogenomics*, (0).
98. Saini, G., Swahn, M. H., & Aneja, R. (2021, March). Disentangling the coronavirus disease 2019 health disparities in African Americans: biological, environmental, and social factors. In *Open Forum Infectious Diseases* (Vol. 8, No. 3, p. ofab064). US: Oxford University Press.
99. Sakuraba, A., Haider, H., & Sato, T. (2020). Population difference in allele frequency of HLA-C* 05 and its correlation with COVID-19 mortality. *Viruses*, *12*(11), 1333
100. Sattar, N., & Valabhji, J. (2021). Obesity as a Risk Factor for Severe COVID-19: Summary of the Best Evidence and Implications for Health Care. *Current Obesity Reports*, 1-8.
101. Schimmel, J., Vargas‐Torres, C., Genes, N., Probst, M. A., & Manini, A. F. (2021). Changes in alcohol‐related hospital visits during COVID‐19 in New York City. *Addiction (Abingdon, England)*.
102. Shoily, S. S., Ahsan, T., Fatema, K., & Sajib, A. A. (2021). Disparities in COVID-19 severities and casualties across ethnic groups around the globe and patterns of ACE2 and PIR variants. *Infection, Genetics and Evolution*, *92*, 104888.
103. Smith, M., Abdesselem, H. B., Mullins, M., Tan, T. M., Nel, A. J., Al-Nesf, M. A., ... & Blackburn, J. M. (2021). Age, Disease Severity and Ethnicity Influence Humoral Responses in a Multi-Ethnic COVID-19 Cohort. *Viruses*, *13*(5), 786.
104. Somogyi, E., Csiszovszki, Z., Molnár, L., Lőrincz, O., Tóth, J., Pattijn, S., ... & Tőke, E. R. (2021). A Peptide Vaccine Candidate Tailored to Individuals' Genetics Mimics the Multi-Targeted T Cell Immunity of COVID-19 Convalescent Subjects. *Frontiers in genetics*, *12*.
105. Strizova, Z., Smetanova, J., Bartunkova, J., & Milota, T. (2021). Principles and challenges in anti-COVID-19 vaccine development. *International Archives of Allergy and Immunology*, 1-11.
106. Sultana, F., & Reza, H. M. (2020). Are SAARC countries prepared to combat COVID-19 to save young, working-age population?. *AIMS public health*, *7*(3), 440.
107. Taylor, K. (2020). mSphere of Influence: that’s racist—COVID-19, biological determinism, and the limits of hypotheses. *Msphere*, *5*(5), e00945-20.
108. Vepa, A., Bae, J. P., Ahmed, F., Pareek, M., & Khunti, K. (2020). COVID-19 and ethnicity: a novel pathophysiological role for inflammation. *Diabetes & Metabolic Syndrome: Clinical Research & Reviews*, *14*(5), 1043-1051.
109. Wakabayashi, M., Pawankar, R., Narazaki, H., Ueda, T., & Itabashi, T. (2021). Coronavirus disease 2019 and asthma, allergic rhinitis: molecular mechanisms and host–environmental interactions. *Current Opinion in Allergy and Clinical Immunology*, *21*(1), 1-7.
110. Walker, A. S., Pritchard, E., House, T., Robotham, J. V., Birrell, P. J., Bell, I., ... & Pouwels, K. B. (2021). Ct threshold values, a proxy for viral load in community SARS-CoV-2 cases, demonstrate wide variation across populations and over time. *medRxiv*, 2020-10.
111. Wambier, C. G., Goren, A., Vaño‐Galván, S., Ramos, P. M., Ossimetha, A., Nau, G., ... & McCoy, J. (2020). Androgen sensitivity gateway to COVID‐19 disease severity. *Drug development research*, *81*(7), 771-776.
112. Wang, L. Y., Cui, J. J., OuYang, Q. Y., Zhan, Y., Wang, Y. M., Xu, X. Y., ... & Yin, J. Y. (2021). Complex analysis of the personalized pharmacotherapy in the management of COVID-19 patients and suggestions for applications of predictive, preventive, and personalized medicine attitude. *EPMA Journal*, *12*(3), 307-324.
113. Wilke, L. G., Nguyen, T. T., Yang, Q., Hanlon, B. M., Wagner, K. A., Strickland, P., ... & Boughey, J. C. (2021). Analysis of the impact of the COVID-19 pandemic on the multidisciplinary management of breast cancer: Review from the American Society of Breast Surgeons COVID-19 and Mastery registries. *Annals of Surgical Oncology*, 1-9.
114. Wu, P., Ding, L., Li, X., Liu, S., Cheng, F., He, Q., ... & Wang, C. (2021). Trans-ethnic genome-wide association study of severe COVID-19. *Communications biology*, *4*(1), 1-10.
115. Xue, X., Shi, J., Xu, H., Qin, Y., Yang, Z., Feng, S., ... & Liu, J. (2021). Dynamics of binding ability prediction between spike protein and human ACE2 reveals the adaptive strategy of SARS-CoV-2 in humans. *Scientific reports*, *11*(1), 1-12.
116. Yamamoto, N., Ariumi, Y., Nishida, N., Yamamoto, R., Bauer, G., Gojobori, T., ... & Mizokami, M. (2020). SARS-CoV-2 infections and COVID-19 mortalities strongly correlate with ACE1 I/D genotype. *Gene*, *758*, 144944.
117. Younis, J. S., Skorecki, K., & Abassi, Z. (2021). The Double Edge Sword of Testosterone’s Role in the COVID-19 Pandemic. *Frontiers in Endocrinology*, *12*.
118. Zhang, Y., Yang, H., Li, S., Li, W. D., Wang, J., & Wang, Y. (2021). Association analysis framework of genetic and exposure risks for COVID-19 in middle-aged and elderly adults. *Mechanisms of ageing and development*, *194*, 111433.
119. Zhou, Y., Hou, Y., Shen, J., Mehra, R., Kallianpur, A., Culver, D. A., ... & Cheng, F. (2020). A network medicine approach to investigation and population-based validation of disease manifestations and drug repurposing for COVID-19. *PLoS biology*, *18*(11), e3000970.
